# The examination of the relationship between working memory and aggression tendency in terms of self-regulation skills

**DOI:** 10.3389/fpsyg.2025.1729799

**Published:** 2026-01-12

**Authors:** Devlet Alakoç, Ruhigül Turan

**Affiliations:** 1 Department of Child Development, Faculty of Health Sciences, Selcuk University, Konya, Türkiye; 2 Department of Child Development, Faculty of Health Sciences, Tarsus University, Mersin, Türkiye

**Keywords:** aggression tendency, mediating role, preschool children, self-regulation skills, working memory

## Abstract

**Introduction:**

The aim of this study was to investigate the mediating role of self-regulation skills in the relationship between working memory and aggression tendencies in preschool children.

**Methods:**

The study employed a correlational survey design. The sample consisted of 118 children aged five years. The data were collected utilising the Personal Information Form, Self-Regulation Scale, Memory Battery for Preschool Children, and Aggression Tendency Scale. To determine the mediating effect in the proposed mode, Pearson correlation and regression analysis based on the bootstrap method were employed.

**Results:**

The findings showed that working memory was a positive predictor of self-regulation skills and a negative predictor of aggression tendency. Self-regulation skills negatively predicted aggression tendencies. Additionally, selfregulation skills were identified as a mediating factor in the relationship between working memory and aggression tendency.

**Conclusion:**

The findings indicate that self-regulation skills are an influential factor in explaining the relationship between working memory and aggression tendencies in preschool children.

## 1 Introduction

Aggression, as a significant determinant of social behaviors, constitutes an instinct that influences a child’s social development and is present in the individual from birth (Babaroğlu, 2013; Olcay, 2008). The presence of various theoretical perspectives seeking to explain the phenomenon of aggression makes it difficult to establish a single, comprehensive definition of the concept. For a behavior to be characterized as aggression, it must either cause harm to a person or an object, or, even in the absence of negative outcomes, be performed with the intention to cause such harm (Baron and Richardson, 1994; Ballard et al., 2004; Çelikkaleli and Tümtaş, 2017; Kartal and Bilgin, 2009). The cognitive social learning theory emphasizes the critical role of cognitive and social factors by proposing that children’s awareness of their own abilities and the consequences of their actions influences their propensity for aggression, and that aggressive behaviors are shaped through the interaction of learning, expectancy, and perceptual processes (Perry et al., 1986). Another explanatory framework for aggression, the General Aggression Model, offers a comprehensive theoretical structure integrating biological, cognitive, and social factors to understand aggressive behaviors (Anderson and Bushman, 2002). According to the model, aggression emerges as a result of both immediate situational factors and long-term developmental processes. In a given moment, an individual’s personal characteristics interact with the existing environmental conditions and this affects their instantaneous emotions, cognitive appraisals, and level of arousal. This situation leads the individual to give either an impulsive or a deliberate response to the situation. On the other hand, the repetition of these instantaneous events can, over time, alter the information structures in the person’s brain and transform them into a more aggressive personality. In conclusion, the model associates aggression both with immediate triggers and with lifelong learning processes (Allen and Anderson, 2017). In the model, it is emphasized that negative emotions may trigger aggressive behavior; however, these emotions are not always sufficient on their own for aggression to occur. The individual determines how to behave by regulating their negative emotions through cognitive processes such as evaluation and decision-making (Allen et al., 2018).

The literature indicates that children’s abilities to perceive and evaluate aggressive interactions emerge at very early stages of life. For example, the study conducted by Kanakogi et al. (2013) shows that 7–10-month-old preverbal infants can distinguish between prosocial (helping) and aggressive (blocking/chasing) behaviors and that they tend to approach the victim or the helper while avoiding the aggressor. Similarly, Geraci et al. (2024) demonstrated that infants determine these social evaluation preferences not only through motion cues but also by combining movement patterns with emotional facial expressions, and that they tend to approach the victim when these cues are consistent. When these findings are considered together, it appears that this evaluative mechanism observed in infancy may be a natural tendency originating from a biological basis. This view is supported by the finding that even newly hatched chicks with very limited social experience spontaneously avoid individuals that display aggressive or rejecting behaviors (De Roni et al., 2023). This suggests that avoiding aggressive interactions has a biological basis and exhibits phylogenetic continuity across species (De Roni et al., 2023).

Interestingly, the emergence of aggressive behaviors is associated with the development of social–moral evaluation and defense mechanisms. For example, Kanakogi et al. (2017) found that infants approve of and prefer third-party individuals who intervene to defend a victim from an aggressor. This result shows that this ability in infants develops rapidly to include evaluating both the intention and the outcome of the intervention. In addition, it has been found that young children not only form their own moral preferences (Geraci and Simion, 2022) but also begin to understand and apply social norms; they expect victims to orient more toward defenders (Geraci, 2020) and foresee the punishment of those who do not protect individuals in distress (Geraci, 2021). These findings indicate that aggression in early childhood emerges within a more complex social context shaped by moral principles, which influences both children’s aggressive actions and their expectations of prosocial defense.

The preschool period is a stage in which children’s positive behaviors—such as cooperation, communication, taking responsibility, empathy, and self-control—develop through positive experiences and appropriate interventions (Robbins et al., 2022), while, at the same time, aggressive behaviors may also be observed as their communication skills and opportunities increase (Shahaeian et al., 2017). The term “aggression” is defined in two distinct ways in the context of early childhood: physical and relational (Baker, 2022; Khoury-Kassabri et al., 2020; Yıldızbaş and Sak, 2020). Physical aggression involves actions such as physically harming or attempting to harm another individual, as well as pushing, hitting, and biting. Relational aggression refers to the infliction of psychological harm or coercion on another individual through the use of verbal means (Crick and Grotpeter, 1995). Though physical aggression, which emerges in infancy, is used by children as a form of self-expression around age two, the prevalence of such behaviors declines throughout the preschool period. During this period, children are expected to develop the capacity to regulate their behaviors (Tremblay et al., 2004). Nevertheless, it has been documented that children engage in relational aggression as a form of self-expression during this developmental stage (Crick et al., 2006; Côté et al., 2007; Vitaro et al., 2006).

A review of the literature indicates a correlation between aggressive behaviors and various negativ outcomes, including poor social skills, high levels of externalizing problems, peer rejection, low peer acceptance, student-teacher conflict, and adjustment difficulties (Campbell et al., 2006; Evans et al., 2018; Gower et al., 2014). Broidy et al. (2003) investigated the developmental trajectory of physical aggression in childhood and its association with violent or non-violent criminal behaviors in adolescence. They reported that problematic behaviors continue from childhood through adolescence, often developing into physical aggression. A study conducted by Brame et al. (2001) found that children with higher tendencies toward physical aggression were more likely to exhibit higher levels of aggressive behavior in adolescence. Examining the short- and long-term effects of aggressive behaviors, along with related risk factors and their persistence, is a significant research priority. A comprehensive understanding of the risk factors associated with aggression is considered essential for the development of effective interventions in this field. Recognizing early aggression symptoms and designing preventive interventions are crucial for reducing the risk of violent behaviors later in life (Tremblay et al., 2004; Côté et al., 2006). Emphasizing the physical, social, and cognitive components in interventions to prevent aggression is an effective strategy for reducing the long-term effects (Richard et al., 2023; Torregrosa et al., 2020), which guided the focus of the present study. Given the evidence that aggression is influenced by emotional regulation and cognitive processes (DeWall et al., 2011), this study was designed to examine the effects of self-regulation and working memory skills on aggressive behaviors.

A core component of cognitive processes (Karakelle and Ertuğrul, 2012; İvrendi, 2020), working memory is a contributing factor to the elevated incidence of aggressive behaviors in young children (Demeusy et al., 2018; Barnett et al., 2001). It is defined as a cognitive system that allows children to encode, store, and retain the information necessary for completing a task (Diamond, 2013; Doğan, 2011; Gözüm, 2020). Working memory plays a pivotal role in resolving social conflicts and promoting social skills, as it allows individuals to assess situations from the perspective of others (Benavides-Nieto et al., 2017). It is noteworthy that aggressive behaviors in children are associated with the social information processing process (De Castro, 2004). This process is a theoretically grounded social–cognitive model that focuses on the cognitive steps occurring from the moment social input is received until a response is generated. The model consists of the stages of encoding social cues, interpreting these cues, determining the goal, searching for possible responses, evaluating these responses, and implementing the behavior. The healthy and accurate functioning of this process contributes to adaptive social behaviors, whereas biased, incomplete, or erroneous processing may lead to the emergence of problematic social responses (Ziv and Elizarov, 2019; Vasconcellos et al., 2006; Crick and Dodge, 1994). In this context, impairments in working memory may increase the likelihood of aggression by negatively affecting the social information processing. Impaired working memory can lead to misinterpretations and aggressive reactions by weakening the ability to accurately encode, interpret, and respond to social cues (Van Rest et al., 2018). Children exhibiting aggressive behaviors process social information differently from their peers. These children encode and represent information in a distinctive way, and they also produce more aggressive responses by focusing less on social targets and selecting more aggressive responses from the options available (Horsley et al., 2010). Nevertheless, children with well-developed working memory and emotion recognition skills tend to use a broader range of information that extends beyond the immediate context. Consequently, impairments in emotion recognition, interpretation, working memory, and inhibition may elevate the risk of aggressive responses in social information processing and problem-solving (Van Nieuwenhuijzen and Vriens, 2012). A study by Rohlf et al. (2018) examined four cognitive skills – working memory, inhibition, cognitive flexibility, and planning – within the context of executive functions. The researchers investigated the relationship between these skills and aggressive behaviors, and found that working memory contributed more significantly to the model than the other cognitive skills. A robust working memory facilitates children’s ability to regulate emotions and manage aggressive tendencies. As working memory capacity increases, aggressive reactions tend to decrease (Juujärvi et al., 2006), indicating a strong associated between aggression a working memory. In studies examining the relationship between children’s working memory and aggression behaviors across various developmental periods, including the preschool years, it was observed that children with lower working memory performance exhibited higher levels of aggressive behaviors (Granvald and Marciszko, 2016; McQuade et al., 2013; Poland et al., 2016; O’Toole et al., 2019; Jakubovic and Drabick, 2020). In contrast, other studies have suggested that high working memory capacity may be associated with increases in goal-directed and physical aggression behaviors (Hecht and Latzman, 2018; Hepditch, 2018). The discrepancies indicate that additional variables may influence the relationship between working memory and aggression.

In this context, research examining the relationships among self-regulation skills, working memory and aggression (Hofmann et al., 2012; Tozduman Yaralı and Güngör Aytar, 2017) highlights self-regulation skills as a key variable. Self-regulation skills play a key role in enabling children to manage and control their emotions, thoughts, and behaviors (Blair and Diamond, 2008). These skills are defined as the ability to organize thoughts and emotions as required and to adapt goals and reactions flexibly according to changing conditions (Woltering and Shi, 2016). According to Vygotsky’s socio-cultural theory, self-regulation represents the transformation of basic biological processes into higher psychological functions through social interaction and instruction (Diaz et al., 1990; Bodrova et al., 2011). In this process, self-regulation skills are acquired as children internalize social rules and behavior control by using self-guiding speech within their “zone of proximal development” and through the role of language as a psychological tool (Vallotton and Ayoub, 2011; Williford et al., 2013). Bandura describes self-regulation as the ability to independently control cognitive and behavioral processes such as managing attention, retaining information, planning and executing a task, and sustaining motivation. This skill involves guiding one’s thoughts, behavior, and motivation according to self-determined standards. The process includes stages such as controlling attention, organizing and executing tasks, monitoring one’s own performance, and adjusting behaviors based on self-evaluation (Bandura, 1991; O’Malley, 2005). Within this context, aggression is often associated with a lack of self-control, and there is a substantial body of literature on this topic. A lack of self-control is associated with increased aggression, whereas strong self-control is associated with decreased aggression. Neuroscience studies elucidate the relationship between self-control and aggression from a neurological perspective, emphasizing the importance of enhancing self-control in regulating aggressive behaviors (Denson et al., 2012). Studies also showed that children’s difficulties in coping with negative emotions, such as disappointment, are linked to deficits in self-regulation. This may contribute to increased aggressive responses. Therefore, it is important to enhance children’s regulatory skills and address these issues through early interventions (Calkins and Dedmon, 2000; Olson et al., 2011). A review of the literature reveals a negative correlation between self-regulation skills and aggressive behaviors (Eisenberg et al., 2010; Polat and Göncü, 2021; Raaijmakers et al., 2008; Valiente et al., 2007; White et al., 2013). Additionally, self-regulation skills have been identified as a key factor in reducing children’s aggressive behaviors during early childhood (Clark et al., 2021). A study carried out by Posner and Rothbart (2000) found that deficiencies in impulse control underlie aggressive behaviors. These deficiencies are caused by poor self-regulation skills and cognitive processes. Improvements in aggressive behaviors have been observed as self-regulation skills develop.

In this context, understanding the origins of aggression and the mitigating its harmful effects serves as a significant focus for research and intervention (Cho, 2013; Yamamoto et al., 2023; Zheng and Zhang, 2016). Previous research reported correlations among working memory, self-regulation skills, and aggression tendencies (Granvald and Marciszko, 2016; McQuade et al., 2013; Poland et al., 2016; O’Toole; 2019; Jakubovic and Drabick, 2020; Eisenberg et al., 2010; Polat and Göncü, 2021; Raaijmakers et al., 2008; Valiente et al., 2007; White et al., 2013). Further research, particularly with the preschool-aged children, is needed to gain a deeper understanding of the precise nature of these variables and their interrelationships. In this context, the present study aimed to examine the relationships among working memory, aggression, and self-regulation skills, as well as investigate the mediating role of self-regulation skills in the relationship between working memory and aggression. The study initially focused on the correlation between working memory, self-regulation skills and aggression tendency. It then examined the effect of working memory on self-regulation skills, and finally, explored the mediating role of self-regulation skills in the working memory and aggression relationship.

## 2 Methods

### 2.1 Research model

**Figure 1:**
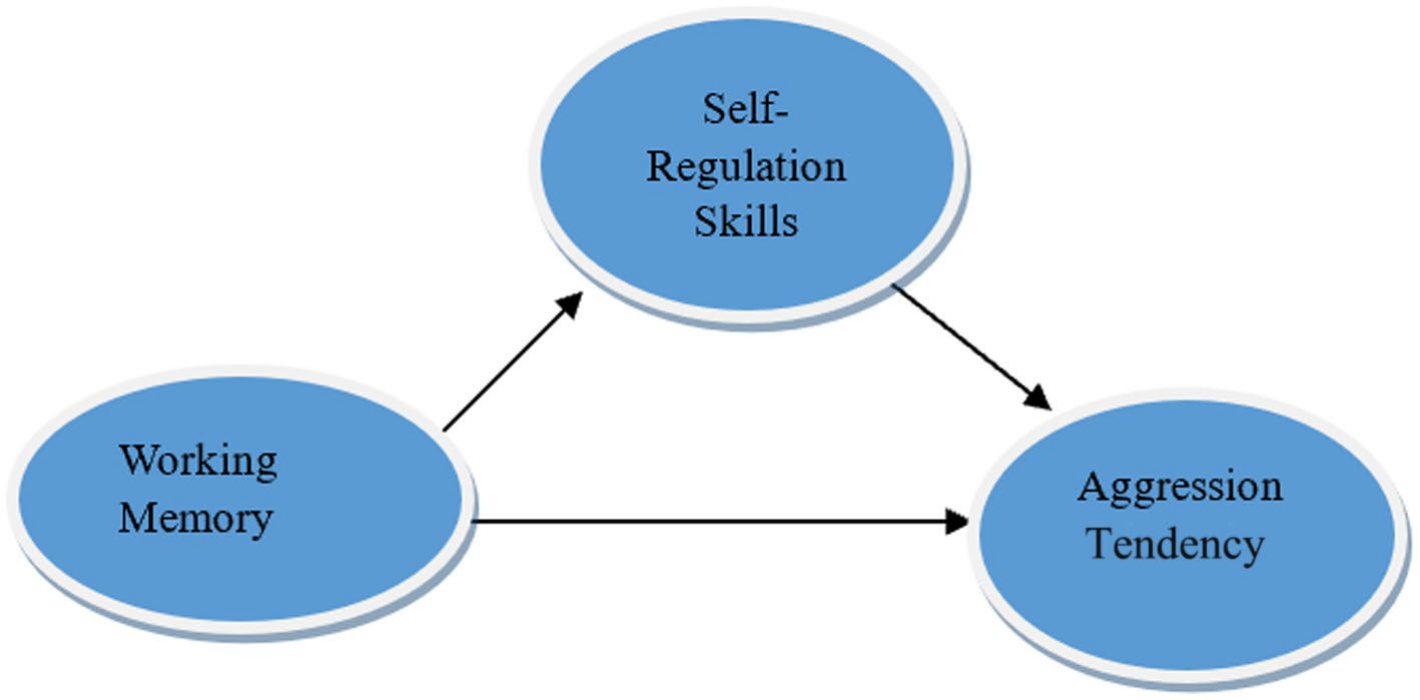
Model illustrating self-regulation skills as a mediator between working memory and aggression tendencies.

This study employed a correlational survey design, which aims to examine the relationships among multiple variables and how these relationships manifest (Karasar, 2015). To explore the mediating role of self-regulation skills, the mediating effect was analyzed using the Bootstrap method. The model illustrating the mediating role is given in Figure 1.

In examining cure 1, it is first essential to investigate the influence of working memory (the independent variable) on self-regulation skills (the mediating variable). Next, the effect of self-regulation skills on aggression tendencies is examined, followed by an analysis of the direct effect of working memory on aggression tendencies. In this study, the independent variable is preschool children’s working memory, the dependent variable is their aggression tendency, and the mediating variable is self-regulation skills. Mediation models allow researchers to examine effects that are not directly observable in the relationship between the independent and dependent variables (Baron and Kenny, 1986; Yılmaz and Dalbudak, 2018).

### 2.2 Participants

The study included 118 children aged five years who were enrolled in independent kindergartens and preschools affiliated with the Ministry of National Education in Konya. Of these participants, 55 (46.6%) were male and 63 (53.4%) were female. Regarding sibling status, 36 children (30.5%) were only children, 51 (43.2%) had one sibling, and 31 (26.3%) had two or more siblings. The mean age of mothers was 32 years, and the mean age of fathers was 35 years. Concerning parental education, 52 mothers (44.1%) had completed high school, while 66 (55.9%) held a university degree. Among fathers, 36 (30.5%) had completed high school, and 82 (69.5%) held a university degree. The research sample was selected using the convenience sampling method, which allows researchers to rapidly and efficiently obtain a sample from a suitable population while considering cost and time constraints (Yıldırım and Şimşek, 2006).

### 2.3 Data collection tools

Personal Information Form: This form includes questions regarding the age, gender, and number of siblings of the participating children, as well as the age and educational status of their parents.

Self-Regulation Scale (Teacher Form): Developed by Bayındır and Ural (2016), this scale is designed to assess self-regulation skills in children aged 48 to 62 months. It comprises 33 items rated on a 5-point Likert scale, organized into two dimensions: regulation skills and control skills. Validity and reliability studies identified 21 items for the regulation skills and 12 items for the control skills. The internal consistency coefficients were 0.96 for regulation skills, 0.91 for control skills, and 0.96 for the entire scale (Bayındır and Ural, 2016). In the context of the present study, the Cronbach’s *α* reliability coefficient was 0.93.

Memory Battery for Preschool Children: Obalı (2018) developed a battery to assess three dimensions of working memory in children aged 61 to 72 months: phonological, visual–spatial, and semantic memory. The visual–spatial and phonological subscales evaluate working memory skills, while the semantic memory subscale assesses semantic memory. The visual–spatial memory consists of three subtests, each using distinct visual stimuli, including black-and-white matrices, colored matrices, and shaped matrices. Each subtest is divided into five sections, with ten tasks per section. Children are first shown the relevant matrices for five seconds to observe, examine, and memorize them. Subsequently, they are asked to select the appropriate squares from small boxes and place them in the correct positions in the empty matrix. The number of correctly placed squares is recorded, and the total score is calculated from all sections. The phonological memory consists of 21 nonsense words presented verbally, which the child is expected to repeat. One point is awarded for each correct repetition, and no points are given for incorrect responses. Content validity was established through expert consultation. In the visual–spatial memory, item-total correlation values for the black-and-white matrices ranged from 0.30 to 0.78 (Cronbach’s α = 0.91), for the colored matrices from 0.22 to 0.76 (Cronbach’s α = 0.91), and for the shaped matrices from 0.26 to 0.79 (Cronbach’s α = 0.93). For the phonological memory, the discrimination values of the 21 items ranged from 0.30 to 0.51, with a KR-20 reliability coefficient of 0.74 (Obalı, 2018).

Aggression Tendency Scale: The scale, developed by Kaynak et al. (2016), is designed to assess the nature and extent of aggression tendencies in children aged 36 to 72 months. It includes 27 items rated on a 7-point Likert scale and is organized into four dimensions: physical aggression toward others, relational aggression toward others, aggression toward oneself, and aggression toward objects. In the original validity and reliability studies, the Cronbach’s α internal consistency coefficient was 0.95 for the entire scale and 0.94, 0.94, 0.85, and 0.92 for the respective sub-dimensions (Kaynak et al., 2016). In the context of the present study, the Cronbach’s α reliability coefficient was calculated as 0.88.

### 2.4 Data collection and analysis

Prior to data collection, ethical approval was obtained from the Selçuk University Faculty of Health Sciences Non-Interventional Clinical Research Ethics Committee. Following this, the directors of the independent kindergartens and nursery schools were interviewed and informed about the research process. Subsequently, Subsequently, parents completed a consent form that outlined the study’s purpose, procedures, and participants’ rights. Before participation, volunteer children were introduced to the researcher to establish a trust-based relationship. The children were informed about their role in the study and their right to withdraw at any time, and their verbal consent was obtained. Various strategies were employed to ensure children’s comfort and encourage their active engagement and autonomy in the participation process. The procedure was discontinued for any child who appeared bored or uninterested. The researcher greeted each child a distraction-free room, where all necessary materials were prepared in advance and engaged in brief, friendly conversation to promote relaxation before the assessment began. The ‘Aggression Tendency Scale’ and ‘Self-Regulation Scale’ were completed by the teachers, while the ‘Memory Battery for Preschool Children’ was administered individually by the researcher.

The data were analyzed using the SPSS 26 statistical software package. Firstly, correlations between the variables were analyzed. To ensure the suitability of the data, the following factors were taken into consideration: sample size, the presence of extreme value, the multicollinearity, and the normality assumption. These are prerequisites for the structural equation model (Ullman, 2013). Later, the bootstrap method was employed to test the mediation model. This method enhances the reliability of the results by resampling from the original data for the purpose of modeling numerous regression equations (Preacher et al., 2007; Zhao et al., 2010). In the present study, we employed the bootstrap technique to evaluate the indirect effects of mediator variables using a sample size of 5,000 resamples. The absence of a zero value within the 95% confidence interval for the mediating variables indicates that the indirect effect is statistically significant. Given that the bootstrap method yields more reliable results, it was preferred for testing the mediation model. A *p*-value below 0.05, as recommended by Preacher and Hayes (2008), was considered statistically significant.

## 3 Results

Table 1 shows a statistically significant positive relationship between working memory and self-regulation skills (rho = 0.455 *p* < 0.001), and a statistically significant negative relationship between working memory and aggression tendency (rho = −0.430 p < 0.001). In addition, a statistically significant negative relationship was found between self-regulation skills and aggression tendency (rho = −0.482 p < 0.001).

**Table 1:** The Pearson correlation values indicating the relationships between self-regulation skills, aggression tendency and working memory skills.

Variables	Working memory skills	Self-regulation skills
Self-regulation skills	***r =* 0.455**; ***p <* 0.001**	
Aggression tendency	***r =* −0.430**; ***p <* 0.001**	***r =* −0.482**; ***p <* 0.001**

r, Pearson correlation coefficient, the sections highlighted in bold are statistically significant (*p* < 0.05).

As evidenced by the results presented in Table 2, there is a statistically significant positive correlation between working memory and self-regulation skills, with a *β* coefficient of 0.46 and a p-value less than 0.001. Furthermore, working memory accounts for 20.7% of the variance in self-regulation skills, a finding that is statistically significant (*F* = 30.298, *p* < 0.001). In the mediator model, working memory demonstrated a negative, statistically significant effect (*β* = −0.27 ± 0.05, *p* = 0.003) on aggression tendency, while self-regulation skills exhibited a negative, statistically significant effect on aggression tendency (*β* = −0.36 ± 0.06, *p* = 0.001). The combined effect of working memory and self-regulation skills on aggression tendency was found to be statistically significant, with an explained variance of 28.8% (*F* = 23.282, *p* < 0.001).

**Table 2:** The findings regarding to the mediating model of self-regulation skills in the effect of working memory on aggression tendency.

Forecast variables	Outcome variables			
	Self-regulation skills		Aggression tendency	
	*β* ± se	*P*	*β* ± se	*p*
Working memory skills	0.46 ± 0.07	**<0.001**	−0.27 ± 0.05	**0.003**
Self-regulation skills	-	-	−0.36 ± 0.06	**<0.001**
Stable	76.43 ± 8.49	**<0.001**	86.50 ± 7.65	**<0.001**
	***R***^**2**^ **= 0.207**		***R***^**2**^ **= 0.288**	
	***F =* 30.298**; ***p <* 0.001**		***F =* 23.282**; ***p <* 0.001**	

*β*, regression coefficient; se, standard error; *R*^2^, coefficient of determination, sections highlighted in bold are statistically significant (*p* < 0.05).

**Figure 2:**
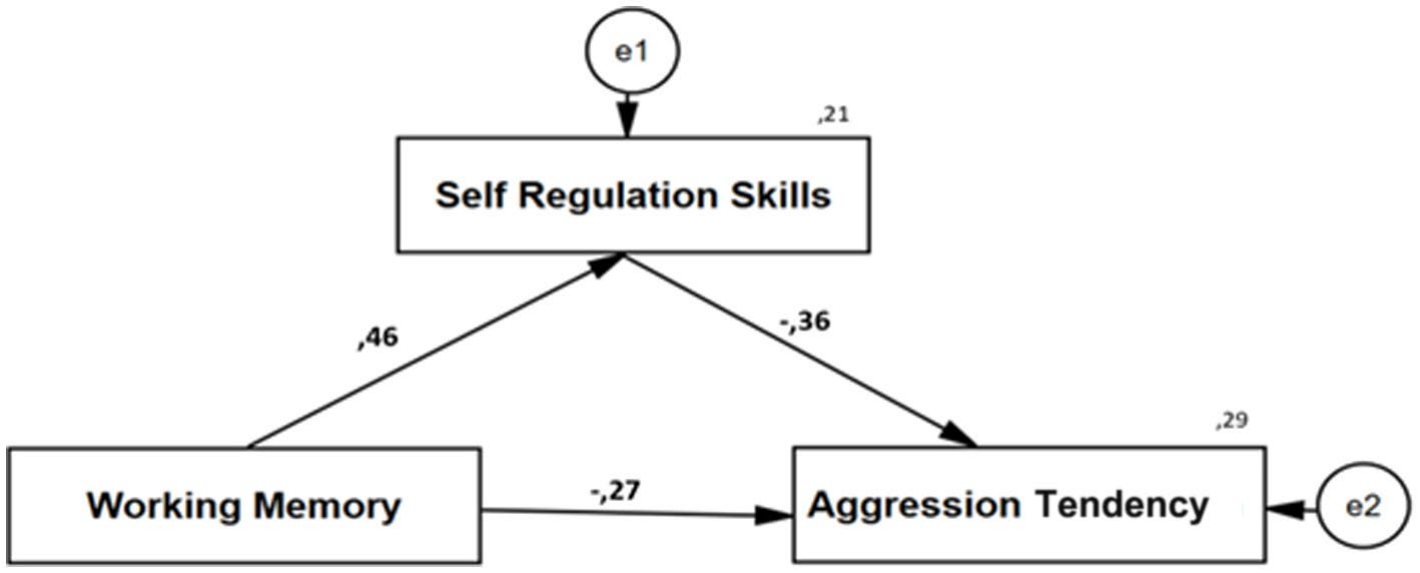
Model illustrating the mediating effect of self-regulation skill on the relationship between working memory on aggression tendency.

Table 3 presents the results of the bootstrap analysis, which investigates the mediating role of self-regulation skills in the relationship between working memory and aggression tendency. An analysis of the direct effects showed that working memory exerted a significant negative effect on aggression tendency, decreasing it by 0.16 ± 0.05 units (*p* = 0.003). The findings regarding the mediating effect indicate that the indirect effect of working memory on aggression tendency through self-regulation skills was statistically significant [Bootstrap Coefficient = −0.16, 95% G.A. = (−0.260, −0.053)]. These results confirm the mediating effect of self-regulation skills. However, while the direct effect of working memory on aggression tendency was 0.16, this effect decreased to 0.10 when the mediating variable was included in the model. The direct effect was also statistically significant (0.003 < 0.05), indicating that self-regulation skills exerted a partial mediating influence. The model developed for this purpose is illustrated in Figure 2.

**Table 3:** Bootstrap confidence levels for mediating variable effect.

Effects	***β*** ± se	***p***	95% Confidence interval for *β*	
			Lower limit	Upper limit
İndirect Effect	−0.10 ± 0.03	**0.005**	−0.179	−0.044
Total Effect	−0.25 ± 0.05	**<0.001**	−0.352	−0.156
Direct Effect	−0.16 ± 0.05	**0.003**	−0.260	−0.053

*β*, regression coefficient; se, standard error; sections highlighted in bold are statistically significant (*p* < 0.05).

It was therefore determined that improvements in working memory skills contributed to the enhancement of self-regulation abilities, and that simultaneous increases in both working memory and self-regulation were associated with a significant decrease in children’s aggression tendencies.

## 4 Discussion

This study investigated the interrelationships between working memory, aggression tendencies, and self-regulation skills in preschool children, with a particular focus on the mediating role of self-regulation skills in the relationship between working memory and aggression tendencies. The findings of our research indicated that working memory was negatively correlated with aggression tendency. These findings suggest that higher levels of working memory skills in children are associated with lower aggression tendencies, whereas lower working memory skills may be linked to increased aggression tendencies. Working memory is a cognitive system that allows for the temporary storage and processing of information necessary for complex cognitive tasks, such as language comprehension, learning, and reasoning. It is also a crucial component in interpretation processes that require maintaining awareness of previous events and integrating them with subsequent occurrences. In this context, working memory is responsible for a number of processes, including the processing, mental reordering, organization, transformation of instructions into action plans, and updating of information. The formation of relationships, evaluation of alternatives, mental association of information, and reasoning processes facilitate the integration of past memories and anticipated future events into planning and decision-making processes (Diamond, 2013; Baddeley, 1992). In this context, impaired memory and attention functions have been linked to misinterpretation of social cues, ultimately leading to adverse behavioral consequences (Crick and Dodge, 1996). A study by Kofler et al. (2011), involving children diagnosed with attention deficit hyperactivity disorder (ADHD) and a comparison group of typically developing children, found an indirect effect of working memory deficits on social problems. The meta-analysis study conducted by Schoemaker et al. (2013) on preschool children demonstrated a relationship between executive function skills (working memory, inhibition, cognitive flexibility) and externalizing behavior problems. In studies conducted by McQuade et al. (2013), Jakubovic and Drabick (2020), and Pahlavan et al. (2012), a relationship was identified between poor executive functioning skills and aggressive tendencies. Additionally, lower working memory performance was associated with peer rejection, poor general social competence, physical, relational, proactive, and reactive aggression, and impaired conflict resolution skills. In a separate investigation conducted by Granvald and Marciszko (2016), a negative correlation was identified between working memory and various forms of aggression (overt, relational, reactive, and proactive). The significance of executive function skills in understanding aggression in children was highlighted. The findings support our conclusion that working memory influences aggression tendency.

The second finding of our study indicated that self-regulation skills were negatively correlated with aggression tendency. This result suggests that children with higher self-regulation skills demonstrate lower aggression tendencies, while children with lower self-regulation skills exhibit higher aggression tendencies. White et al. (2013) reported a correlation between reactive aggression and deficient behavioral regulation and executive functioning. Tozduman Yaralı and Güngör Aytar (2017) discovered that children with inadequate self-regulation skills tend to display aggression, rule-breaking, and disruption of group dynamics, as well as other behavioral problems. These children frequently exhibit behavioral problems, including lack of perseverance, difficulty following instructions, and an inability or unwillingness to assume responsibility. The studies conducted by Saraç et al. (2021b) and Robson et al. (2020) revealed a negative correlation between self-regulation skills and several behavioral outcomes, including aggressive behaviors, internalization and externalization problems, peer bullying, asocial behaviors, hyperactivity-distraction, and fear-anxiety levels. In a longitudinal study conducted by Olson et al. (2011), it was seen that children demonstrating higher levels of aggressive interactions with peers exhibited lower self-regulation skills. Another study conducted by Brajša-Žganec and Hanzec (2015) indicated that children with higher self-regulation skills and a strong understanding of emotions exhibited the least aggressive behaviors, whereas those with lower self-regulation skills and less effective emotion understanding displayed the highest levels of aggression. The results of this study support our findings regarding the effect of self-regulation skills on aggression tendency.

Our study also found a positive correlation between working memory skills and self-regulation skills. Working memory is defined as a system that allows an individual to process, maintain, and recall information in alignment with their goals (Unsworth and Engle, 2007). This system encompasses storage, re-storage, and attention control mechanisms that are essential for information processing (Chein and Schneider, 2005). Self-regulation skills are defined as the process of reaching one’s goals through the monitoring and adjustment of one’s behavior (Marqués et al., 2005). The two concepts share numerous common points, as both working memory and self-regulation skills are associated with an individual’s ability to direct attention and process information. In particular, when faced with demanding or complex circumstances, individuals are compelled to deploy both working memory and self-regulation skills effectively (Kane and Engle, 2002). However, the relationship between working memory and self-regulation also shows some differences. Although working memory is concerned with the temporary storage and processing of information, self-regulation skills focus on how individuals can regulate their behaviors based on this information. Furthermore, while self-regulation processes assist in directing an individual’s attention toward specific goals, working memory provides the cognitive resources necessary for the execution of these processes (Schmeichel, 2007; Vohs and Baumeister, 2004). Consequently, working memory and self-regulation skills are complementary processes, yet working memory enables the processing of information, and self-regulation is responsible for using this information to achieve desired outcomes (Ilkowska and Engle, 2010; Unsworth and Engle, 2007). In addition, while self-regulation skills involve adaptive modifications to internal states, emotions, thoughts, or actions, executive function skills include cognitive processes that enable the effective implementation of these self-regulation skills when (Nigg, 2017). Schmeichel et al. (2008) investigated the correlation between individual differences in working memory capacity and the regulation of emotional expressions and emotional experiences. Their findings indicated that individuals with higher working memory capacity demonstrated improved efficacy in regulating their emotional reactions. Graziano et al. (2015), Saraç et al. (2021a), and Tarle et al. (2019) reported that working memory plays an essential role in self-regulation skills, with working memory performance influencing emotion regulation and expression skills in children. These findings align with our research results.

Our study indicated that self-regulation skills have a mediating effect on the relationship between working memory and aggression tendency. An increase in children’s working memory skills enhances their self-regulation abilities, and the increase in both skills, in turn, reduces children’s aggression tendencies. Accordingly, it can be suggested that self-regulation and working memory skills play an important role in reducing aggression tendencies in children. This finding is considered to be consistent with the Social Information Processing (SIP) Model, which explains how children process social situations. The model includes the steps of encoding and interpreting social cues, determining the goal, searching for and evaluating possible responses, and implementing the behavior. The accurate execution of these steps contributes to adaptive social behaviors, whereas errors or deficiencies in the process may lead to the emergence of undesirable social responses (Ziv and Elizarov, 2019; Vasconcellos et al., 2006; Crick and Dodge, 1994). In the study conducted by Caporaso et al. (2021), working memory was identified as the component most strongly associated with the SIP steps, and children with low working memory were found to experience difficulties in social competence during peer conflicts and to be more prone to aggression. Impaired working memory results in an inability to accurately encode and interpret social cues, which can subsequently lead to misjudgments and thus aggressive responses (Van Rest et al., 2018). In contrast, a strong working memory enables children to regulate their emotional responses and control their aggressive tendencies (Juujärvi et al., 2006). The regulation and adjustment of emotions are skills that require control, including self-regulation (Eisenberg et al., 2010). Self-regulation is an important skill for children to anticipate the potential outcomes of a behavior and to control aggression. According to social–cognitive approaches, the mental structure that contains beliefs about the consequences of specific behaviors is closely related to predictions regarding potential outcomes. These beliefs have a determining influence on which behavior the child will select and implement in the subsequent steps of the Social Information Processing (SIP) model (Lochman, 2010). In this context, it is considered that working memory contributes to the prevention of aggressive behaviors by enhancing the effectiveness of self-regulation skills and supporting emotional control processes. Posner and Rothbart (2000) emphasized that children’s inability to regulate their impulses is a fundamental factor underlying aggressive behaviors. This phenomenon can be attributed to deficiencies in self-regulation skills and cognitive processes. Conversely, the gradual development of self-regulation skills has the potential to mitigate aggressive behaviors. In the study conducted by Holley et al. (2017) examined the relationship between emotion regulation, executive function skills, and aggressive behaviors, as well as their reciprocal effects. The findings indicated that students with lower emotion regulation and executive function skills were the group most at risk for aggressive behaviors. The research further demonstrated that strong executive function and emotion regulation skills serve as protective factors against students’ tendencies to exhibit aggressive behaviors. Furthermore, the results suggest that interventions targeting the enhancement of emotion regulation and executive function skills may be effective in reducing aggressive behaviors among students. These findings are consistent with the results of our research.

## 5 Conclusion and recommendations

The results demonstrated that self-regulation skills play a mediating role in the relationship between working memory and aggression tendency. Specifically, an enhancement in preschool children’s working memory skills led to an improvement in their self-regulation skills, while the concurrent development of both skills resulted in a reduction in their aggression tendency. Within this framework, the study is considered significant for identifying both the individual and combined effects of working memory and self-regulation skills on children’s aggression tendencies. The findings of the study corroborate those of previous research, which have elucidated the relationship between working memory, self-regulation skills, and aggression tendency. Moreover, the present study has expanded our knowledge in this field by revealing the mediating effect of self-regulation skills. Our findings will contribute to the process of determining the goals and content of early intervention programs to be prepared by emphasizing the importance of working memory and self-regulation skills in preventing children’s aggression tendencies in preschool period. Training programs for preschool teachers could focus on enhancing their understanding of how working memory and self-regulation skills—both independently and in combination—affect aggression, and how these competencies can be effectively fostered within classroom settings. For future research, to achieve a more comprehensive understanding of preschool children’s aggression behaviors, it would be beneficial to explore additional social and emotional factors that may influence these behaviors, as well as to investigate other potential mediating or moderating factors associated with cognitive processes.

## 6 Limitations

This study was conducted with a sample of five-year-old children. The results can be extended to other age groups through cross-sectional or longitudinal studies. This will facilitate a more expansive research scope and the attainment of findings that are more generally valid. A further limitation of the study is that the assessment of children’s aggression tendency and self-regulation skills was conducted by teachers alone. In light of these considerations, future studies may assess children’s aggression tendencies and self-regulation skills through direct observation or by using measurement tools specifically designed for children.

## Data Availability

The raw data supporting the conclusions of this article will be made available by the authors, without undue reservation.
